# Flow-Assisted Corrosion and Nondestructive Testing of Multi-Medium Transmission Pipelines: A Review

**DOI:** 10.3390/ma19112272

**Published:** 2026-05-27

**Authors:** Boran Cui, Guangwei He, Fangchao Kang, Gaoshen Cai, Shuqian Shen, Haozhe Jin

**Affiliations:** 1 School of Mechanical Engineering, Zhejiang Sci-Tech University, Hangzhou 310018, China; 2 School of Energy and Power Engineering, Guangdong University of Petrochemical Technology, Maoming 525000, China

**Keywords:** multi-medium transmission pipelines, flow-assisted corrosion, multiphase flow, nondestructive testing, ultrasonic guided wave, deep learning

## Abstract

The aim of this review is to clarify the mechanism and influencing factors of flow-assisted corrosion in multi-medium transmission pipelines for pipeline safety management, along with the progress in nondestructive testing in this vein. Such pipelines undergo severe flow-assisted corrosion under multiphase-flow, high-temperature, high-pressure, and complex chemical conditions, threatening structural integrity and operational safety. This study summarizes the dominating roles of aqueous wetting, mass transfer, and flow-induced shear stress in corrosion evolution and analyzes the coupling effects of hydrodynamics, medium chemistry, and material properties on corrosion deterioration. The applicational advantages and limitations of ultrasonic guided-wave, magnetic flux leakage, and eddy current testing in corrosion detection are systematically concluded. Future development trends combining artificial intelligence, machine learning, and digital twins are projected, providing a reference for intelligent detection and full-life-cycle integrity management of transmission pipelines.

## 1. Introduction

In recent years, oil-and-gas-pipeline construction has expanded rapidly in China, with the total pipeline mileage expected to reach 240,000 km by 2025. As the most efficient, economical, and stable means of transportation, pipelines have formed a nationwide “west-to-east and south-to-north” layout. Nevertheless, pipeline corrosion readily leads to damage, leakage, and rupture, with corrosion-related failures accounting for 25% of oil-and-gas equipment faults and attracting widespread attention in regard to safety [1,2,3,4]. Three authoritative industrial standards lay the foundation for pipeline corrosion research: API 571 [5] classifies flow-assisted corrosion and other failure modes; ASME B31.3 [6] governs corrosion design and material selection for high-pressure, high-temperature multiphase pipelines; and ISO 23221 [7] standardizes full-life-cycle corrosion monitoring and integrity management in regard to pipelines.

This review has a clearly defined research scope: the formation mechanism of, factors influencing, and nondestructive detection technologies applied to flow-assisted corrosion (FAC) in multi-media transmission pipelines under multi-phase flow conditions. As a typical multi-field coupled failure mode, flow-assisted corrosion’s formation and development involve phenomena discussed in a number of disciplines, including fluid mechanics, corrosion electrochemistry, and materials science, making FAC an interdisciplinary topic. Compared with static corrosion, FAC is characterized by a high corrosion rate, complex forms of damage, diverse influencing factors, and significant coupling effects, posing enormous challenges regarding the accurate detection and evaluation of pipeline corrosion conditions. Along with traditional destructive testing methods, such as cutting sampling inspection, the hydraulic pressure burst test, the slow strain rate test, metallographic microstructure analysis, and corrosion product component analysis, electrochemical detection technologies, including open-circuit potential, polarization curve, and electrochemical impedance spectroscopy, have also been widely adopted in the evaluation of pipeline corrosion. Electrochemical methods can be used to quantitatively characterize the corrosion kinetic process, interfacial charge transfer behavior, and microscopic corrosion mechanisms of pipeline steel and have become an important auxiliary means of analyzing the evolution law of flow-assisted corrosion. These methods have been widely applied. However, they usually require well-trained professionals or expensive testing equipment, and the testing process requires pipeline shutdown, posing numerous restrictions and challenges in practical engineering applications [8,9,10,11,12]. As a testing means that does not damage the structural integrity of pipelines, nondestructive testing (NDT) technology enables in situ, real-time, and quantitative detection of flow-assisted corrosion defects in pipelines, serving as the key technical support for solving corrosion safety control problems pertaining to multi-medium transmission pipelines [13,14,15]. Therefore, systematically sorting out the research progress on NDT technology in this field, deeply analyzing the advantages and shortcomings of existing technologies, and clarifying future research directions are of great practical and long-term significance for promoting academic innovation and engineering applications in related fields.

In recent years, domestic and foreign scholars have carried out extensive research on flow-assisted corrosion (FAC) of multi-medium transmission pipelines and obtained phased research results. Experimental and numerical studies have confirmed that aqueous-phase wetting is the primary route whereby FAC is induced; in oil–water and oil–gas–water multiphase-flow service environments, pipeline surface material shedding and localized corrosion thinning are jointly controlled by crude oil properties, flow velocity, phase state, water cut, pipeline material, and structural disturbance of pipeline fittings [16,17]. Meanwhile, relevant research on pipeline defect detection and safety evaluation has been continuously deepened. Nondestructive testing (NDT), a non-damaging detection method, has become the core technical means of pipeline corrosion and defect assessment. NDT methods such as radiographic testing, ultrasonic testing, visual inspection, eddy current testing, magnetic particle testing, and other conventional methods have been widely applied and verified in engineering [18,19,20,21,22,23]. Scholars have also carried out exploratory research on the integrated application of NDT detection data and corrosion prediction models. However, obvious research gaps still exist: the multi-field coupling mechanism of FAC under complex multi-medium conditions has not been fully clarified; there is a lack of research systematically comparing the applicability of various NDT methods under actual working conditions; and the integrated application of NDT data and corrosion prediction models is still in the exploratory stage.

Within the overall research context, this study logically links corrosion mechanisms, material characteristics, and nondestructive testing applicability to form a coherent discussion. Based on the research status noted above and existing limitations, this study systematically summarizes the formation mechanisms and main influencing factors of flow-assisted corrosion in multi-medium transmission pipelines under multiphase flow conditions. The conventional nondestructive testing methods commonly used for pipeline defect inspection are categorized, and the status of research on combining detection data with prediction models is analyzed. The existing research deficiencies are summarized, providing a clear reference for further in-depth research on the flow-assisted corrosion mechanism and targeted selection of nondestructive testing technologies in pipeline engineering.

## 2. Mechanism of Flow-Assisted Corrosion in Multi-Medium Transmission Pipelines

Corrosion is defined as the spontaneous physicochemical degradation of metallic materials induced by continuous interaction with surrounding gaseous, liquid, or soil environments, resulting in surface deterioration, microstructure degeneration, and a gradual reduction in structural service performance. For buried steel pipelines used for oil, gas, and multiphase fluid transportation, corrosion occurs through a combined internal and external electrochemical mechanism. Externally, variations in soil resistivity, oxygen concentration, and groundwater salinity form differential corrosion cells that trigger outer-wall material dissolution. Internally, the water phase contained in multiphase media wets the inner pipe surface, while dissolved CO_2_, H_2_S, and chloride ions drive persistent anodic dissolution. Coupled with flow shear stress inside the pipeline and long-term soil electrochemical attacks outside, this synergistic process gradually causes wall thinning and local structural damage. As a typical localized corrosion behavior of buried pipeline steel under service conditions, pitting corrosion usually initiates at surface defects or inclusions or on incomplete passive films. Aggressive chloride ions in soil and transported media easily adsorb and break down the local protective layer, forming tiny, closed corrosion pits. Inside the pits, solution hydrolysis reduces local pH and accelerates metal dissolution, producing a self-catalytic propagation effect. The oxygen concentration difference inside and outside the pit sustains a stable galvanic reaction, driving the pit to develop inward continuously. Under the combined action of multiphase flow disturbance and medium corrosion, pitting gradually expands and becomes a major hidden danger for pipeline perforation and leakage.

### 2.1. Multiphase Flow-Dominated Corrosion Mechanism

Multiphase flow is the core scenario regarding flow-assisted corrosion in gathering and transportation pipelines in the petroleum industry, and it triggers corrosion through a complex physicochemical process induced by aqueous-phase wetting of the steel surface, through which materials detach from the pipeline’s surface. The presence of water is a necessary condition for corrosion initiation, and once the steel surface is wetted by water, localized corrosion will result in thinning of the pipe walls [24].

Multiphase flow affects the corrosion process through three key interrelated mechanisms. The first mechanism is phase distribution and wettability. Aggregation of droplets in oil–water-dispersed phases, phase interface fluctuations, and droplet/bubble impingement on the pipe wall will trigger abrupt changes in local corrosive medium concentrations and shear stress, forming corrosion acceleration zones. When the aqueous phase is dispersed in the oil phase in the form of droplets without wetting the pipe wall, the corrosion risk is significantly reduced, whereas when the aqueous phase forms a continuous phase or accumulates at the bottom of the pipeline, the corrosion scope and rate greatly increase. The second mechanism is mass transfer. Radial mixing and eddies under turbulent flow accelerate the mass transfer of corrosive media (such as CO_2_, H_2_S, and Cl^−^) to the pipe wall and promote the discharge of corrosion products, while mass transfer under laminar flow relies on molecular diffusion, and the degree of corrosion is relatively mild. A high mass transfer rate will hinder the formation of protective corrosion product films or accelerate their detachment [25]. The third mechanism is flow-induced corrosion. Shear stress generated by high flow velocity destroys the integrity of corrosion product films (such as FeCO_3_ and FeS), triggering a “corrosion-erosion” cycle [26]. Notably, the corrosion rate does not always rise monotonically with an increase in flow velocity and shear stress; such promotional effects are strongly constrained by flow regime and practical operating conditions. Under laminar flow conditions at low velocity, weak wall shear maintains the integrity of corrosion product films, and the limited mass transfer rate restrains electrochemical reaction intensity, resulting in a relatively low and stable corrosion level. In a transitional flow state, fluctuating shear stress alternately damages and rebuilds surface passive films, leading to unstable localized corrosion. By contrast, a moderate turbulent flow can accelerate the renewal of corrosive media, yet an excessively high flow velocity in some supercritical CO_2_ or high-temperature working environments may facilitate the formation of dense and stable passive films instead, thereby inhibiting further corrosion. Additionally, operating parameters such as system temperature, pressure, water cut, and medium composition jointly determine the critical threshold of flow shear accelerating corrosion. For instance, high water cut under slug flow aggravates the local impact and thinning, while low water cut with a dispersed oil–water flow reduces wall wetting and suppresses corrosion even at a relatively high flow rate.

**Figure 1:**
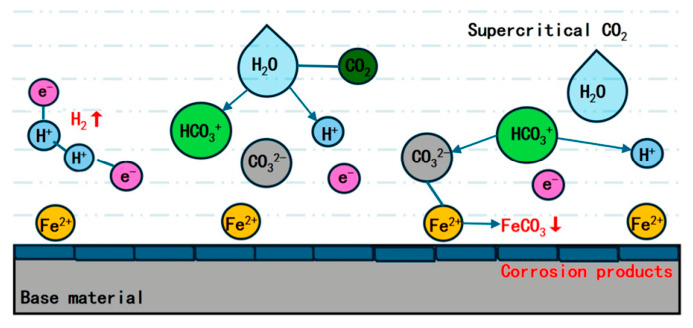
Schematic diagram of corrosion [27].

Flow disturbances at local structures such as pipeline elbows and valves form eddies, leading to increased local shear stress; meanwhile, eddies impede the discharge of corrosion products, further exacerbating localized corrosion. Flow-related corrosion phenomena are summarized in Figure 1.

The properties of crude oil indirectly regulate corrosion by affecting the characteristics of multiphase flow: The density difference between crude oil and water affects the accumulation state of the aqueous phase. When their densities are similar, oil and water are difficult to separate, reducing the risk of localized corrosion; the higher the viscosity of crude oil, the easier it is to entrain the aqueous dispersed phase, thereby reducing the wetting of the pipe wall by water. Components such as asphaltenes and paraffins in crude oil can adsorb onto the pipe wall to form a protective film and mitigate corrosion, but this film is vulnerable to erosion and damage by high-velocity fluids. Phase inversion (IP) is a critical aspect of multiphase flow corrosion. When the water cut reaches a critical value, the water-in-oil (*w/o*) emulsion transforms into an oil-in-water (*o/w*) emulsion, and the continuous aqueous phase leads to comprehensive wetting of the pipe wall, resulting in a sharp increase in the corrosion rate [28,29].

Based on the existing research on the electrochemical mechanism of FAC and the corresponding corrosion medium action, an academic consensus has been reached on the basic coupling law of fluid shear and electrochemical reaction, as well as the independent corrosion mechanisms of CO_2_, H_2_S, chloride ions, and microorganisms. However, obvious research divergences still exist in regard to the synergistic corrosion mechanism of multiple corrosive media under multiphase flow conditions, the critical concentration threshold of Cl^−^ inducing localized corrosion, and the interaction between microbial metabolism and flow shear. Most studies focus on single-factor static corrosion analysis, while the multi-factor coupling effect under actual oil–gas–water multiphase flow still lacks systematic induction and unified conclusions, and corrosion failure characteristics are rarely linked with the technical requirements of subsequent nondestructive testing.

### 2.2. Flow-Induced Electrochemical Corrosion Mechanism

There are multiple forms of corrosion in pipeline service environments, mainly including flow-assisted corrosion (FAC), CO_2_ corrosion, H_2_S corrosion, chloride-induced localized corrosion, and microbiologically influenced corrosion (MIC). Although these forms of corrosion often coexist and interact in multiphase media, they differ significantly in terms of dominant driving factors, physical processes, and electrochemical mechanisms. The differences among various pipeline corrosion forms are summarized in Table 1.

The electrochemical aspect of flow-assisted corrosion lies in the coupling of fluid mechanics and electrochemical corrosion, and fluid flow regulates corrosion kinetics by altering the conditions of electrode reactions. Corrosive gases (CO_2_ and H_2_S), dissolved salts (Cl^−^ and SO_4_^2−^), and microorganisms in the corrosive medium are the core triggers of electrochemical corrosion [30,31]. CO_2_ dissolves in the aqueous phase, forming carbonic acid, which reduces the pH value of the medium and induces hydrogen depolarization corrosion. The protectiveness of its corrosion product, FeCO_3_, depends on conditions such as temperature and pressure: a dense and adherent protective film can be formed at 50–70 °C, while a film formed at low temperatures is transparent, with a thickness of less than 1 μm [32,33]. H_2_S ionizes, producing H^+^ and S^2−^ after dissolution; H^+^, as a strong depolarizer, accelerates the anodic dissolution of Fe, and the generated products, such as FeS, can form a corrosion product film, but some hydrogen atoms will penetrate the steel matrix and trigger hydrogen embrittlement.

Dissolved salts accelerate charge transfer by increasing the conductivity of the medium. Cl^−^ can penetrate the passivation or corrosion-product film to induce pitting corrosion, and the increase in Cl^−^ concentration will enhance the autocatalytic effect of pitting corrosion [34,35]. Dissolved oxygen induces localized corrosion through oxygen concentration cells, causing severe corrosion when the concentration exceeds 0.1 mg/L; there is a critical concentration value, beyond which the corrosion rate decreases with an increase in concentration. The core mechanism of microbiologically influenced corrosion (MIC) is the alteration of the local corrosion environment by metabolites (such as H_2_S) of microorganisms, including sulfate-reducing bacteria (SRB). Meanwhile, the synergistic effect between the biofilm, the metal surface, and corrosion products accelerates electrochemical corrosion [36,37,38], and the synergism between SRB and iron-oxidizing bacteria (IOB) further intensifies pitting corrosion.

Flow conditions regulate the corrosion rate by affecting the mass transfer process of electrochemical reactions: At low flow velocities, the limited oxygen diffusion makes the cathodic reaction the rate-controlling step, resulting in a low corrosion rate. At high flow velocities, oxygen mass transfer is accelerated, boosting the cathodic reaction rate; meanwhile, shear stress destroys the corrosion product film, leading to a significant increase in the anodic dissolution rate. In a supercritical CO_2_ environment, the strong shear stress generated by high flow velocities can prevent the formation of porous and non-protective corrosion layers, instead promoting the generation of a dense passivation film and inhibiting corrosion.

### 2.3. Pipeline Material Composition and Characteristic Properties

Oil-and-gas multi-medium transmission pipelines are mainly manufactured using low-carbon micro-alloyed pipeline steels such as X52, X65, X70, and X80, which have become mainstream structural materials for long-distance gathering and transportation pipelines due to their excellent mechanical properties, weldability, and comprehensive corrosion resistance.

In terms of chemical composition, pipeline steel is predominantly made up of carbon (C) and manganese (Mn). A suitably low carbon content can effectively improve weldability and avoid the creation of a brittle microstructure, while manganese plays a role in solid solution strengthening and grain refinement. Trace elements such as silicon (Si), phosphorus (P), and sulfur (S) are strictly controlled in industrial production. Excess P and S will obviously reduce toughness and induce segregation and inclusion defects, further aggravating localized corrosion. Alloying elements such as chromium (Cr), molybdenum (Mo), and nickel (Ni) are often added to improve the service adaptability of pipeline steel under high-temperature, high-pressure, and medium corrosive conditions. Cr can promote the formation of dense passive films such as Cr(OH)_3_ and FeCO_3_ films, while Mo and Ni help inhibit sulfide stress corrosion and improve structural stability in H_2_S-containing environments.

In terms of basic mechanical properties, conventional pipeline steels possess high yield strength, low-temperature toughness, and good plastic deformation capacity, allowing them to adapt to complex ground settlement and internal pressure fluctuations in long-distance transportation. Excellent weldability ensures on-site construction quality and reduces corrosion sensitivity at weld joints. With respect to material microstructure, hot-rolled pipeline steel is mainly composed of ferrite and pearlite or acicular ferrite. A uniform and fine-grained structure can significantly improve resistance to flow-induced shear damage and electrochemical corrosion, while an uneven microstructure and grain boundary segregation are likely to trigger localized FAC thinning.

Along with the multiphase-flow and complex-medium service background of multi-medium transmission pipelines discussed in this review, the correlation between material characteristics and flow-assisted corrosion is prominent. Chemical composition and microstructure directly determine the intrinsic corrosion resistance of the pipe matrix in CO_2_/H_2_S and chloride-containing environments. Alloy element content affects the compactness and stability of corrosion product films such as FeCO_3_ and FeS under flow shear. Pipeline steel with poor microstructural uniformity and high impurity content is more likely to form local high-risk corrosion areas under the combined action of a multiphase flow pattern impact, water cut changes, and sand particle erosion. Moreover, mechanical strength and surface hardness also determine material resistance to erosion–corrosion coupling damage under long-term high-speed multiphase flow conditions. Therefore, pipeline material composition and inherent properties are important internal controlling factors coordinating with hydrodynamic and medium chemical factors to jointly dominate the initiation and development of flow-assisted corrosion.

### 2.4. Section Summary

Different corrosion mechanisms lead to a number of different defects, such as uniform thinning, local pitting, and erosion-corrosion damage. These different defect characteristics impose different requirements on the detection performance and working-condition adaptability of nondestructive testing technologies, providing a clear basis for the subsequent comparative analysis of NDT methods.

## 3. Nondestructive Testing Technologies for Flow-Assisted Corrosion in Multi-Medium Transmission Pipelines

Nondestructive testing (NDT) technologies are widely applied to evaluate pipeline conditions. Common methods include ultrasonic testing, magnetic flux leakage testing, eddy current testing, radiographic testing, and the use of penetrant liquids. Each method has its own advantages and limitations. After years of development and optimization, these technologies have seen remarkable improvements in detection accuracy, anti-interference capacity, automation level, and other aspects and have been widely used in pipeline corrosion detection under various working conditions. The following section will systematically review the detection principles, application status, and optimization directions of various traditional NDT technologies in flow-assisted corrosion detection, focusing on analyzing their advantages and limitations under multi-medium working conditions.

### 3.1. Ultrasonic Testing Technology

**Figure 2:**
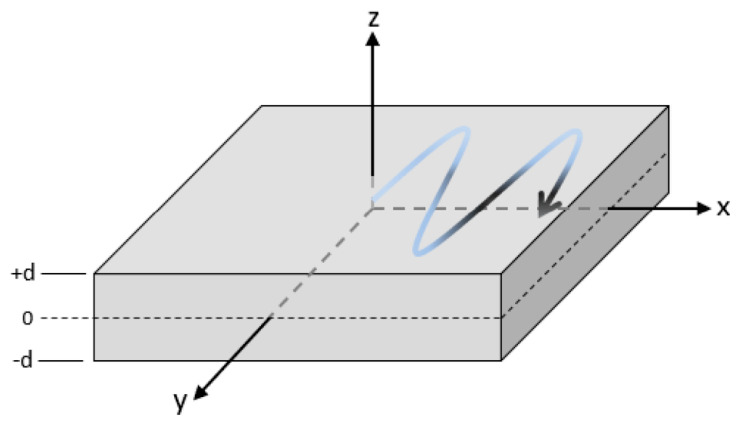
Commercial guided propagation in SH wave mode, illustrated by the gradient arrow, in a plate with a thickness of 2*d*, where the propagation is along x and the material particle displacements are along y [42].

Ultrasonic guided-wave testing is an emerging NDT method. Guided waves are mechanical waves that propagate in waveguide structures, which are widely used in the nondestructive testing of plate-like and tubular structures [39]. Guided waves have broad applications in the NDT of plate and tubular components. They can propagate along pipelines over long distances without full scanning, greatly improving detection efficiency, and are particularly suitable for pipelines in inaccessible areas, such as buried and overhead pipelines. During testing, specific types of guided waves are excited, and waveform reflection and conversion will occur when defects are encountered. Based on this phenomenon, the location and size of defects can be inversely deduced, so these waves are commonly used for the early defect screening of oil and gas pipelines. Pipeline defect screening based on guided waves provides a reliable reference for formulating maintenance plans according to pipeline operating conditions, thereby enhancing the effectiveness of maintenance strategies [40,41]. Abuassal, A, et al. [42] systematically summarized the modal mechanism, transmission and reception principles, transducer design, engineering applications, existing challenges, and future development trends of unidirectional ultrasonic guided-wave technology for nondestructive testing and evaluation. In the section concerning ultrasonic guided-wave modes, the propagation characteristics of shear horizontal (SH) waves are elaborated, and a corresponding physical propagation model is presented in Figure 2.

**Figure 3:**
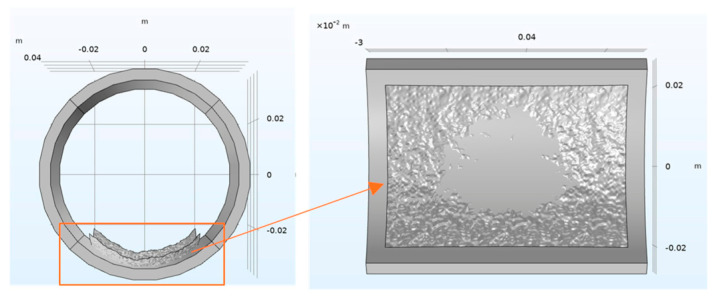
Schematic of random surface fitting with a pipeline [49].

Ultrasonic guided-wave testing has become a widely adopted method for buried pipeline detection [43]. Sun YJ et al. [44] conducted research on treated pipelines using ultrasonic guided-wave testing; determined the position, amplitude, and cause of echoes via echo A-scanning; and integrated wavelet analysis to extract the flat echo amplitude from the original signal, thus more accurately detecting defects with small-amplitude echoes. Aiming to address the difficulty of quantitatively evaluating defect size in pipeline corrosion screening when using long-distance ultrasonic guided-wave testing, Mudge PJ et al. [45] developed a method for defect size characterization by focusing ultrasonic energy with phased array transducers. The results show that this technology can provide a preliminary basis for assessing defect severity without requiring additional testing. Raisutis R et al. [46] developed a method for detecting corrosion defects in steel pipes using helical-mode ultrasonic guided waves. Through finite element modeling and experimental analysis, it was found that corrosion defects with different depths and sizes can induce changes in the phase delay of the amplitude peak of helical guided-wave signals, thus verifying the feasibility and potential of pipeline NDT based on phase delay differences. Zhang PD et al. [47] studied the application of quasi-distributed acoustic sensors (q-DAS) in pipeline structural damage identification. Combined with simulation and experimental calibration, synthetic q-DAS data were generated and verified under active ultrasonic guided-wave conditions. The results show that the calibrated simulation model accurately reflected the key signal characteristics of defects such as corrosion and welding irregularities and maintained a good degree of consistency with actual measurement results. Targeting the difficulty of preparing delamination defect samples from carbon fiber composites, Jing Z et al. [48] carried out ultrasonic testing research based on finite element simulation, established an ultrasonic testing model based on the pulse reflection method (as shown in Figure 2), and used COMSOL Multiphysics 6.3 to develop a delamination defect model, which was experimentally verified. The results demonstrate that the simulated and measured signals are highly consistent in terms of amplitude and time-domain positioning, with an error of less than 3%, verifying the reliability and economy of this method in the quantitative detection of delamination defects. Li, Y, et al. [49] proposed a curved random surface-modeling method based on spatial frequency composition, established a finite element model of pipeline internal corrosion that closely represented the real morphology, and quantitatively evaluated corrosion damage in layered pipelines by combining ultrasonic guided-wave technology and a wavelet packet decomposition algorithm. The fitting effect of the random surface on the inner wall of the pipeline, along with the structure of the pipeline, is shown in Figure 3.

**Figure 4:**
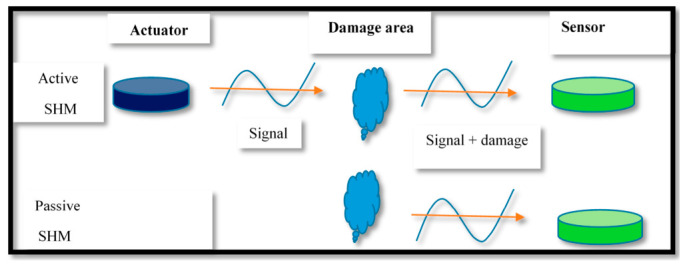
Active and passive SHM methods [55].

In addition, El Mountassir et al. [50] collected an ultrasonic guided-wave signal database over a long duration under different temperature conditions, aiming to determine the influence of ambient temperature on ultrasonic guided-wave propagation and false-alarm defects. Corrosion-like defects were introduced at the end of the monitoring period to verify the effectiveness of temperature compensation methods and damage detection algorithms in structural health monitoring (SHM). Teoh CY et al. [51] investigated the response characteristics of torsional mode T(0,1) ultrasonic guided waves in pipeline corrosion defects, established mathematical and numerical models of pipeline corrosion defects, and revealed that asymmetric corrosion can induce a conversion to flexural mode F(1,m) and that the reflection coefficient increases monotonically with defect depth, providing a basis for symmetry discrimination and precise positioning in regard to corrosion defects. Lv LL et al. [52] proposed a deep neural network method based on physical model embedding to efficiently quantitatively reconstruct pipeline wall thickness using ultrasonic guided-wave signals. The results show that this method can accurately invert the position, shape, and depth of corrosion defects with high resolution and precision, achieving a speed improvement of about 300 times relative to traditional full-waveform inversion while ensuring an average inversion accuracy of approximately 87–89%, and the feasibility of real-time quantitative imaging was verified through experiments. Zhang Y et al. [53] systematically studied the propagation and scattering mechanisms of ultrasonic guided waves in marine pipeline elbows, revealed the modal structure and scattering characteristics of T(0,1) mode at elbows via a semi-analytical finite element method and numerical simulation, established a theoretical framework for interpreting elbow guided-wave signals, and experimentally confirmed that T(0,1) mode is highly sensitive to defects on the back sides of elbows. Ling JT et al. [54] proposed an improved ultrasonic guided-wave damage imaging method incorporating the local signal difference coefficient (LSDC) and reliability coefficient (RC) to address the difficulty of characterizing local signal changes via the traditional RAPID algorithm and ameliorate its susceptibility to noise interference, thereby realizing more reliable identification of the locations of pipeline corrosion. The experimental results show that the overlap rate between predicted defects and actual defect locations reached 73.02–79.43%. Abbas M et al. [55] reviewed the applications of ultrasonic guided waves (UGWs) in defect diagnosis and health monitoring regarding metal structures. Structural health monitoring (SHM) is a comprehensive technical system that integrates sensing, signal analysis, and damage identification. It is designed for real-time online monitoring, early warning, and health status evaluation in regard to engineering structures and has been widely applied in pipeline corrosion defect detection and long-term safety service assessment. Structural health monitoring includes active and passive methods. Schematics of active and passive structural health monitoring methods are shown in Figure 4. They reveal that there is still a lack of in-depth research on the shape and orientation of defects in plate-like structures and experimental research, combined with numerical simulation, exploring the influence of cracks with different shapes and orientations on ultrasonic guided-wave responses, indicating a need to establish an empirical model framework to identify these structural defects.

Ultrasonic guided-wave testing leverages guided waves’ capacity to propagate long distances in waveguide structures, thereby allowing rapid overall screening of structures such as pipelines, and it offers distinct advantages in inaccessible areas, including buried and overhead pipelines. By analyzing the reflection, scattering, and mode conversion of guided waves at defects, researchers can locate and preliminarily quantitatively evaluate types of damage such as corrosion and cracks in pipelines, providing technical support for analysis of pipeline operation status and decision-making regarding maintenance.

Extensive work has been conducted on guided-wave signal feature extraction, modal propagation mechanisms, and quantitative characterization of defects. Relevant results show that combining wavelet analysis, phased array focusing, and specific guided-wave modal analysis methods helps to improve detection sensitivity for micro-defects and complex corrosion defects. Studies based on finite element simulations and experimental verification further reveal the propagation and scattering laws of guided waves in different structures and under different defect conditions. Meanwhile, deep learning constrained by physical models significantly improves imaging efficiency while ensuring high quantitative accuracy.

Overall, the extent of the application of ultrasonic guided-wave testing in pipeline defect screening and structural health monitoring is relatively mature, but there are still deficiencies in the aspects of defect morphology, directional influence, and environmental factor interference under complex structural conditions. Follow-up research can further improve the stability and quantitative reliability of detection results based on the integration of guided-wave mechanism analysis and data-driven methods.

### 3.2. Magnetic Flux Leakage Testing Technology

**Figure 5:**
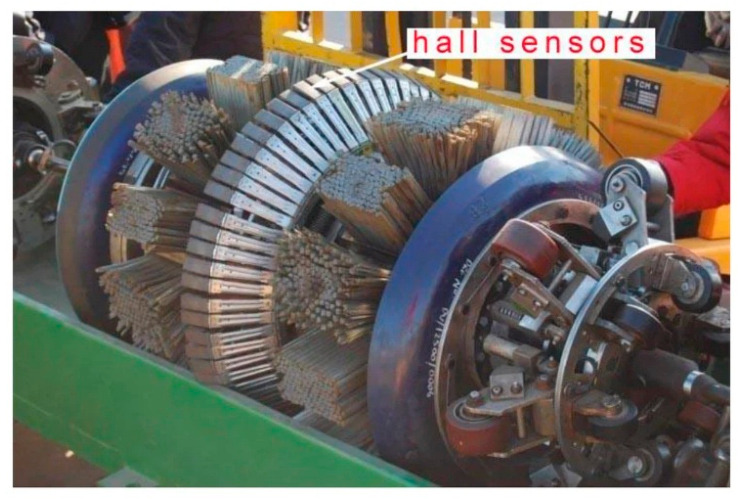
Photo of a magnetic pig with Hall sensors [58].

The pipeline inspection industry has been developing magnetic flux leakage (MFL) technology since the 1960s [41,56,57]. In the initial stage, magnetic particles were widely used in sensors to display results through accumulation. This method is intuitive, simple, and highly sensitive and has been widely applied in industry. With the continued development of the semiconductor electronics industry, significant progress has been made in regard to magnetic sensors, thereby eliminating the need to restrict magnetic particles on measuring tools. Figure 5 shows a photo of a magnetic pig with the Hall sensors.

Magnetic flux leakage testing has been widely used since the early 1950s. Since then, its use has evolved from qualitative defect identification to quantitative analysis [23], with numerous researchers conducting in-depth studies. Yang LJ et al. [59] introduced convolutional neural networks to process leakage magnetic field data, aiming to address the large volumes of data and low recognition efficiency in magnetic flux leakage testing for long-distance oil-and-gas pipelines. Combined with imaging, corrosion, and grayscale preprocessing methods used to highlight defect features, intelligent discrimination and imaging of magnetic flux leakage testing images were realized, significantly improving the efficiency of pipeline defect identification. Jiang Q et al. [60] proposed a novel method for corrosion pit defect identification based on pipeline magnetic flux leakage (MFL) testing. By constructing artificial corrosion pits on the pipeline’s surface and introducing an interference elimination algorithm to compensate for the MFL field, the researchers effectively extracted and qualitatively and quantitatively identified corrosion pit defects. Qu F et al. [61] developed a subsea pipeline inspection pig based on magnetic flux leakage (MFL). Three-dimensional finite element analysis was employed to study the influences of defect geometry, lift-off distance, and running speed on MFL signals, and a defect quantitative inversion method based on axial peak–valley values and radial peak spacing was proposed, with its accuracy verified, providing an effective theoretical framework for subsea pipeline corrosion monitoring and integrity management. Wei HT et al. [62] proposed a new residual magnetic flux leakage (RMFL) crack detection method. Utilizing the hysteresis characteristics of pipelines after MFL magnetization, the authors used theoretical analysis, finite element simulation, and experimental research to verify that the RMFL method is superior to traditional MFL in weak crack signal detection. They also proved that integrating the RMFL module into existing MFL systems enables collaborative and efficient detection of corrosion and crack defects. Peng X et al. [63] studied the detection capacity of individual and combined magnetic flux leakage (MFL) tools for pipeline corrosion defects based on the probability of detection (POD) method. They constructed a POD model with corrosion volume and orientation serving as variables and validated it through in-service pipeline data, determining the minimum criteria required to ensure reliable detection of corrosion defects. Yang Y et al. [64] investigated the effect of residual magnetic fields after MFL testing on the corrosion behavior of X52 pipeline steel in simulated soil solution through open-circuit potential, polarization curve, electrochemical impedance spectroscopy, and corrosion morphology analysis. It was found that the magnetic field generally promoted the electrochemical corrosion process, causing a positive shift in corrosion potential, an increase in corrosion current density, and a decrease in charge transfer resistance. The mechanism is mainly related to the influence of the Lorentz force and the Kelvin force on ion migration. Electrochemical detection methods such as open-circuit potential, polarization curve, and electrochemical impedance spectroscopy are important technical means of evaluating pipeline corrosion behavior. They can effectively characterize the electrochemical reaction processes and interfacial charge transfer characteristics of pipeline steel in complex service environments. Relevant studies have confirmed that residual magnetic fields can accelerate the electrochemical corrosion process, manifesting as a positive shift in corrosion potential, increased corrosion current density, and decreased charge transfer resistance. This effect is mainly attributed to the regulation of ion migration by the Lorentz force and the Kelvin force. Electrochemical detection can quantitatively reflect the kinetic laws behind corrosion and the corresponding microscopic mechanisms and serve as an effective complement to traditional nondestructive testing technologies for flow-assisted corrosion assessment of transmission pipelines. Sun LY et al. [65] systematically studied nondestructive testing methods for fluid transmission pressure pipelines, focusing on reviewing and comparing the detection principles, applicable characteristics, advantages, and disadvantages of magnetic flux leakage (MFL) and acoustic emission (AE) online detection technologies. The authors pointed out that it is difficult to achieve complete defect detection using a single method, providing a reference for the rational selection of pressure pipeline testing technologies. Li CJ et al. [66] established a self-magnetic flux leakage (SMFL) model of elliptical cylindrical defects based on the magnetic charge theory. The quantitative relationship between SMFL signals and defect length, depth, and sensor lift-off value in pipeline pitting defects was systematically studied. Experiments verified that the model can invert and estimate defect parameters according to SMFL signals, realizing the upgrading of metal magnetic memory testing from qualitative positioning to quantitative characterization. Guo J et al. [67] designed an intelligent pipeline leakage detector based on nondestructive testing principles. FPGA was adopted to realize 12-bit high-speed multi-channel (up to 1024 channels) data acquisition, combined with ARM large-capacity storage and network transmission to achieve real-time analysis and quantitative identification of detection data, providing a basis for evaluating the safety of pipeline operations and formulating maintenance cycles.

Since its application in pipeline inspection in the mid-20th century, magnetic flux leakage (MFL) technology has evolved from qualitative detection using magnetic particle display to quantitative analysis based on magnetic sensors, becoming one of the important means of pipeline corrosion and defect detection. With the advancement of sensor technology, numerical modeling, and signal-processing methods, MFL testing has been expanded to defect imaging, quantitative inversion, and applicable scenarios.

Thus far, studies have focused on efficiently acquiring magnetic flux leakage signals and defect characterization. The introduction of convolutional neural networks, imaging, and preprocessing methods has improved the efficiency and stability of defect identification under large-scale MFL data. Additionally, systematic research has been conducted on the geometric parameters of and reliability of detecting typical defects such as corrosion pits and cracks based on finite element analysis, magnetic charge models, and the probability-of-detection method. Meanwhile, methods such as residual magnetic flux leakage and self-magnetic flux leakage have expanded the capacity of traditional MFL in weak-defect detection, and progress has been made in regard to hardware systems for multi-channel high-speed acquisition and real-time processing.

Overall, a relatively complete technical system for magnetic flux leakage testing in pipeline corrosion and structural integrity assessment has been developed, but individual detection methods still have limitations under complex working conditions. Collaborative applications combining intelligent algorithms, physical models, and multiple nondestructive testing technologies will be the main direction of development for improving the accuracy and engineering applicability of pipeline defect detection.

### 3.3. Eddy Current Testing Technology

**Figure 6:**
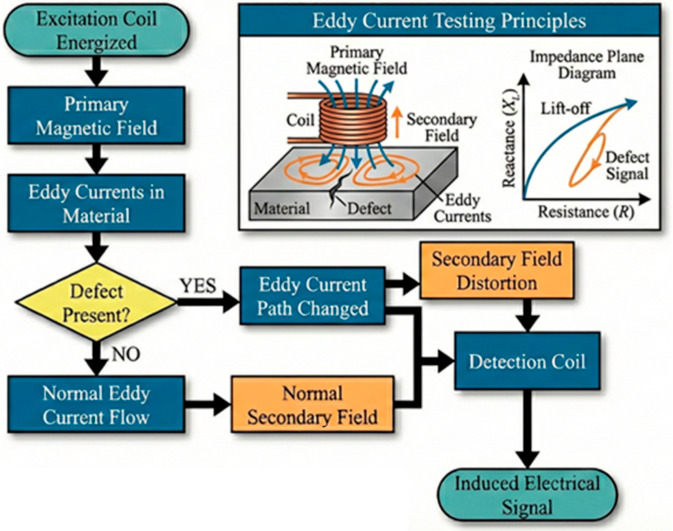
Flowchart of the basic principles of eddy current testing (ECT) [70].

Eddy current testing is based on the interaction between a magnetic field source and a test material. This interaction induces eddy currents in the test material [68]. Extremely tiny cracks can be detected by monitoring the changes in eddy currents. Since the 1950s, eddy current testing has been increasingly critical in material inspection, with prominent applications in aviation, the nuclear industry, and other fields. Extensive research and development regarding highly sensitive eddy current sensors and instruments over the past six decades have established eddy current testing as a widely used inspection technology [69]. Liang, X et al. [70] established a technical system of eddy current in-line inspection for oil-and-gas pipelines and compared the principles, characteristics, and application scenarios pertaining to various eddy current detection methods. A flowchart of the principles of eddy current testing is shown in Figure 6.

Liu HW et al. [71] established a three-dimensional model of pipeline corrosion defects using the finite element method based on the principle of pulsed eddy current nondestructive testing. The variation laws of current, magnetic field, and coil-induced voltage under different defect sizes were systematically analyzed, revealing that magnetic field intensity and current increase with the increase in corrosion depth, while the induced voltage decreases, allowing quantitative detection of internal pipeline corrosion defects. Taheri H et al. [72] employed eddy current array (ECA) nondestructive testing technology to detect and characterize prefabricated notches and stress corrosion cracking (SCC) that had developed under load-corrosion action in steel pipeline materials. The results show that this method can obtain the size and characteristics of SCC defects with high accuracy and repeatability, verifying the effectiveness of ECA in pipeline SCC evaluation. Lai S et al. [73] designed a detection system based on pulsed eddy current technology for the detection of local corrosion in insulated pipeline inner walls. Experimental studies on simulated defects such as large-area thinning, groove corrosion, and pitting corrosion showed that the system can detect wall thickness thinning equal to about 10% even when the thickness of the insulation layer is 110 mm, and the detection accuracy meets the application requirements of pressure pipeline engineering. Yu ZH et al. [74] comparatively studied the focusing characteristics of cylindrical and U-shaped pulsed eddy current (PECT) probes in local corrosion detection under insulation layers. Eddy current dissipation power was introduced to quantitatively characterize focusing performance through simulations and experiments, and the results show that the U-shaped probe had better eddy current-focusing capacity and higher local corrosion detection sensitivity. Gao ZH et al. [75] proposed and verified a non-contact magnetic focusing eddy current testing (MFECT) method for internal inspection of ferromagnetic pipelines. Magnetic focusing was employed to improve the signal-to-noise ratio of receiving coils, weaken the skin effect, and increase penetration depth. The results of simulations and experiments demonstrate that this method exhibits good feasibility and detection performance under optimized operating frequencies. Cornu S et al. [76] systematically reviewed the development and application of eddy-current-testing technology in in-line inspection (ILI). They pointed out that although this technology is mature and has been successfully used to accurately detect surface defects such as pipeline grooves, it has not been commercially applied to ILI tools as a means of crack detection because it is limited to internal wall crack identification. Krysko NV et al. [77] aimed to classify and characterize pipeline surface operational defects. They integrated ultrasonic, eddy current, and computer-vision-based visual inspection methods; collected defect image and measurement datasets; proposed a CNN designed to identify pitting corrosion; and quantitatively measured defect size based on a gradient-boosting model, with the size prediction accuracy of the comprehensive diagnosis algorithm reaching an RMSE of 0.011 mm.

Owing to the eddy current response generated by the interaction between the magnetic field and the tested material, eddy current testing is highly sensitive to tiny cracks and local corrosion and has been widely used in aviation, nuclear power, pipeline inspection, and other fields since the mid-20th century. With the development of eddy current sensors, excitation modes, and signal-processing technologies, this method has gradually evolved from use for qualitative detection to quantitative analysis.

Studies have mainly focused on technical routes such as pulsed eddy current, eddy current array, and magnetic focusing approaches. Relevant work shows that the combination of finite element modeling and experiments can quantitatively reveal the relationship between corrosion defect size and electromagnetic response; eddy current arrays and improved probe structures exhibit good detection accuracy and stability in stress corrosion cracking and under-insulation corrosion detection; and magnetic focusing and non-contact detection methods have certain advantages in improving signal-to-noise ratios, reducing the skin effect, and enhancing penetration capacity. Meanwhile, the application of eddy current testing in in-line inspection is relatively mature, but there are still limitations in crack types and detection depth.

Overall, eddy current testing offers distinct advantages in the identification of local pipeline corrosion and surface defects, but the effect of its application in engineering depends, to a certain extent, on the probe structure, excitation parameters, and signal interpretation methods employed. Integration with other nondestructive testing technologies and intelligent algorithms will help further improve the accuracy and quantitative capacity of defect identification.

### 3.4. Section Summary

Horizontally comparing the three mainstream nondestructive testing technologies summarized above reveals that each method has its own applicable scenarios and inherent limitations. Ultrasonic guided-wave testing is superior in terms of long-distance overall screening and rapid detection of buried pipelines, but it is easily influenced by multiphase flow media and complex pipeline structures and offers limited accuracy in quantitative characterization of small-scale pitting corrosion. Magnetic flux leakage testing is a mature method of identifying volumetric corrosion defects and overall wall thinning suitable for regular in-pipeline inspection, but it is insensitive to tiny cracks and shallow local corrosion. Eddy current testing shows prominent advantages in micro-crack and surface localized corrosion detection, yet its detection depth and anti-interference ability under high-temperature and complex multiphase conditions are insufficient.

## 4. Factors Influencing Flow-Assisted Corrosion in Multi-Medium Transmission Pipelines

Flow-assisted corrosion is a complex process involving the coupling of fluid mechanics and electrochemical corrosion, and the corrosion degree and evolution law are synergistically regulated by various factors. Clarifying the mechanism of action of each influencing factor is the core prerequisite for accurately predicting pipeline corrosion risks, optimizing anti-corrosion design, and ensuring the safe operation of industrial pipeline systems. The system for classifying the factors influencing FAC proposed by the Electric Power Research Institute (EPRI) is widely used in conventional single-phase water–steam pipeline service environments of thermal power plants. According to EPRI guidelines, the factors influencing flow-accelerated corrosion (FAC) can be classified into three categories: hydrodynamics, water chemistry, and component material composition [78]. Nevertheless, faced with the complex oil–gas–water multiphase flow, variable medium components, and sand with the erosion characteristics of petroleum transmission pipelines, the conventional single-phase-oriented classification system cannot fully address the actual corrosion characteristics under field working conditions. On this basis, we take the EPRI classification framework as a basic reference and further supplement and improve the characteristic influencing factors relevant to multiphase-flow corrosion scenarios.

### 4.1. Hydrodynamic Factors

The occurrence and evolution of flow-assisted corrosion are synergistically regulated by multiple factors, and the core influencing factors can be classified into four categories: at the level of fluid flow characteristics, hydrodynamic factors such as flow velocity, flow pattern, erosion angle, and multiphase flow exert effects on flow-assisted corrosion [79,80]; at the level of medium corrosive properties, medium composition, phase state, temperature, pressure, and water cut directly determine the thermodynamic possibility and kinetic rate of corrosion reactions, serving as the internal key to corrosion occurrence [81,82]; at the level of pipeline material properties, the chemical composition, microstructure, and surface state of materials determine the corrosion resistance foundation of the matrix itself, affecting the initiation and development of corrosion reactions; and at the level of pipeline structure and defects, special geometric structures such as elbows and valves, as well as surface defects such as scratches, cracks, and corrosion pits, will induce local flow disturbance and form corrosion acceleration zones, thereby becoming important inducing factors in flow-assisted corrosion [83].

Hydrodynamic factors regulate the FAC rate by affecting the dissolution efficiency of magnetite films and mass-transfer processes, with core parameters including flow velocity, geometric shape, temperature, and mass-transfer characteristics.

Flow velocity: An increase in flow velocity raises the contact volume between iron-free water and the metal surface, accelerating the rate of dissolution of magnetite by expanding the concentration difference, thereby exacerbating FAC damage.

Geometric shape: Complex pipeline structures (such as elbows and straight pipes downstream of orifice plates) induce turbulence and enhance fluid mixing, allowing more iron-free water to make contact with the metal surface and increasing the mass-transfer rate of magnetite from the surface to the mainstream fluid.

Temperature: The FAC rate peaks in the range of 150–180 °C because at this temperature range, the superposition effect of ion solubility is strongest, and the magnetite mass-transfer rate is optimal [84].

Mass-transfer characteristics: The mass-transfer efficiency of soluble ions depends on the Reynolds number (Re), Schmidt number (Sc), and Sherwood number (Sh). The Reynolds number determines the flow regime (laminar, transitional, or turbulent); the Schmidt number reflects the relative thickness of the hydrodynamic boundary layer and mass-transfer boundary layer; and the Sherwood number is a dimensionless expression of the mass-transfer coefficient [85]. The higher the turbulence intensity of the fluid (the larger the Re) and the larger the mass diffusion coefficient (the smaller the Sc), the greater the Sherwood number and the higher the FAC rate.

Flow regime: Different from single-phase working conditions in the conventional EPRI framework, oil–gas–water multiphase pipelines present typical flow patterns, including stratified flow, slug flow, annular flow, and bubbly flow. These flow regimes directly alter phase distribution, wall-wetting conditions, and shear stress distribution. In particular, slug flow has a periodic liquid plug impact and generates local turbulent disturbance, intensifying interfacial mass transfer and destroying the integrity of corrosion product films, thus readily inducing localized wall thinning and aggravated FAC.

Water cut and phase inversion: Water cut is a dominant hydrodynamic parameter for multiphase FAC evolution. When water content exceeds the critical phase-inversion threshold, water-in-oil emulsions convert into an oil-in-water state, leading to continuous aqueous-phase wetting on the inner wall of the pipe and sharply elevating electrochemical corrosion kinetics and FAC severity.

### 4.2. Water Chemistry Factors

Water chemistry factors affect FAC risk by regulating the type and solubility of the protective film on the metal surface, with core indicators including oxidation–reduction potential (ORP) and pH value.

Oxidation–reduction potential (ORP): ORP is a key variable affecting single-phase FAC, and its value determines which type of protective film (magnetite or hematite) will form. When ORP decreases, magnetite films become dominant, significantly increasing the probability of FAC occurrence. However, ORP is affected by factors such as pH and oxygen partial pressure, hindering direct comparison across different power plants.

pH value: pH affects the FAC rate by controlling the solubility product of ferrous hydroxide (Fe(OH)_2_) (the product of Fe^2+^ and Fe(OH)^+^). An increase in pH reduces the formation of the above products, thereby inhibiting magnetite dissolution. In practice, the system pH can be adjusted by adding ammonia or neutralizing amines.

CO_2_ and H_2_S effects: In actual oil–gas–water multiphase transportation systems, CO_2_ and H_2_S are the dominant contributors to medium corrosivity as opposed to only single-phase water chemistry. Dissolved CO_2_ forms carbonic acid, lowering medium pH and driving anodic dissolution of pipeline steel, while the stability of the FeCO_3_ generated is strongly modulated by multiphase-flow shear. H_2_S ionizes, producing corrosive H^+^ and S^2−^, thus accelerating metal dissolution and creating FeS corrosion products; meanwhile, atomic hydrogen penetration may induce hydrogen embrittlement. Multiphase-flow turbulence and interfacial mass transfer further promote the migration of CO_2_ and H_2_S toward the wall of the pipe, producing strong coupling between hydrodynamic behavior and medium chemical corrosion, a phenomenon that cannot be fully explained by the traditional EPRI single-phase FAC classification system.

### 4.3. Component Material Composition

Material composition directly determines the sensitivity of metals to FAC, including matrix material, alloying elements, and material interface effects.

Matrix material: Iron-based materials (such as carbon steel) are prone to FAC, and the addition of alloying elements such as chromium, copper, and molybdenum can significantly reduce the FAC rate [86].

The function of alloying elements: ASME SA-213 T11 alloy, containing 1.25% chromium, and ASME SA-213 T22 alloy, containing 2.25% chromium, have been successfully applied in FAC-sensitive areas. Chromium can promote the formation of amorphous products in the protective film, reducing the oxygen reduction rate during corrosion. The protective film in 3Cr natural-gas transmission pipelines is composed of CrOH_3_ and FeCO_3_, and CrOH_3_ can promote the deposition of FeCO_3_ on the surface of the pipeline, enhancing the anti-corrosion effect [87].

Material interface effects: This includes the “inlet effect” and “reverse effect”. The inlet effect refers to the increase in the difference in the concentration of soluble iron in a fluid when the FAC-resistant material is upstream and the FAC-sensitive material is downstream, accelerating downstream FAC. The reverse effect refers to the turbulence induced by the weld at the material interface when the FAC-sensitive material is upstream and the FAC-resistant material is downstream, leading to aggravated local FAC [88].

Sand erosion coupling effect: Under real-world conditions, oilfield multiphase media inevitably carry fine sand particles. Under high-velocity multiphase flow, solid sand particles continuously impact and abrade the pipeline inner surface, mechanically stripping protective corrosion product films. Combined with electrochemical corrosion induced by CO_2_/H_2_S and an aqueous medium, a synergistic erosion–corrosion acceleration effect occurs. Pipeline steel with poor microstructural uniformity and a high impurity content is more vulnerable to sand impacts and flow shear, further aggravating localized FAC thinning, which is a typical characteristic of multi-medium pipelines beyond the conventional EPRI single-phase FAC framework.

### 4.4. Section Summary

The factors influencing FAC can be categorized into hydrodynamics, water chemistry, and material composition, which function distinctively in corrosion evolution. Hydrodynamic factors, such as flow velocity, flow regime, and water cut, control flow shear and mass-transfer intensity and are highly susceptible to multiphase-flow pattern switching and phase inversion. Water chemistry factors, including pH, ORP, CO_2_, and H_2_S, govern medium corrosivity and the stability of corrosion product films, dominating electrochemical reaction kinetics. Material composition factors (matrix type, alloying elements, and microstructure) determine the inherent corrosion and erosion resistance of pipeline steel, serving as the internal constraint on FAC development.

**Figure 7:**
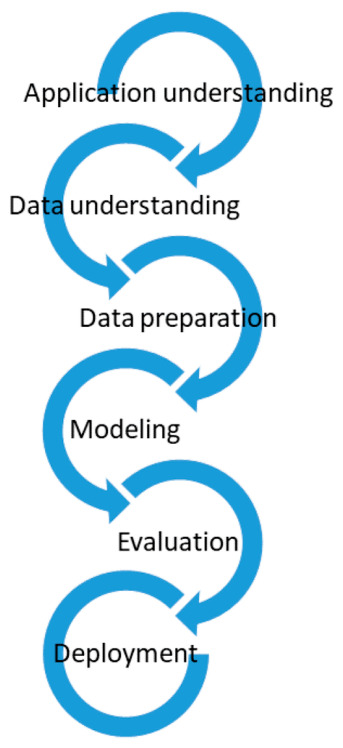
Process model of the application of AI in NDE [89].

Different from the conventional EPRI single-phase framework, multi-medium pipelines are further affected by sand erosion and multiphase coupling effects. The three types of factors jointly (rather than independently) control FAC deterioration, resulting in diverse failure modes, including uniform thinning, pitting, and erosion–corrosion. On this basis, artificial intelligence and machine learning can be further integrated into the optimization of corrosion factor analysis and nondestructive evaluation. A typical process model for intelligent NDE application is shown in Figure 7.

## 5. Conclusions and Prospects

This review systematically covers the formation mechanisms, controlling factors, and progress on nondestructive testing research regarding flow-assisted corrosion (FAC) in multi-medium oil–gas–water transmission pipelines and refines the core academic conclusions of existing studies based on logical induction and horizontal comparison, avoiding redundant repetition of Sections and empty generalized narration. The core research conclusions of this review are summarized as follows:

(1) The primary aspects of FAC in multiphase pipeline services are aqueous phase wetting, phase distribution evolution, and flow-induced shear mass transfer. Different from single-phase static corrosion, the coupling of turbulent disturbance, CO_2_/H_2_S medium dissolution, chloride ion infiltration, and microbial metabolic activity jointly accelerates interfacial electrochemical deterioration, resulting in localized thinning and erosion–corrosion composite failure characteristics unique to oil-and-gas multiphase-flow pipelines.
(2) The classical FAC classification framework proposed by EPRI is mainly applicable to single water–steam working conditions in power plants and cannot be directly extended to complex oil–gas–water multiphase delivery scenarios. Based on the conventional three-factor classification of hydrodynamics, water chemistry, and material properties, flow pattern transformation, critical water cut, and phase inversion effect, as well as sand particle impact erosion, are confirmed to be the additional dominant characteristic factors affecting FAC development in multi-medium pipelines.
(3) Ultrasonic guided-wave, magnetic flux leakage, and eddy current testing have led to relatively mature technical systems for pipeline FAC defect detection, but each has obvious application boundaries. Ultrasonic guided-wave technology is suitable for long-distance overall screening of buried and overhead pipelines; magnetic flux leakage testing offers unique advantages in identifying volumetric corrosion and uniform wall thinning; and eddy current testing is more sensitive to microcracks and shallow localized corrosion. Under high-temperature, high-pressure, and multiphase-medium interference, all three technologies still suffer from signal distortion and decreased quantitative detection accuracy.

In view of the existing research deficiencies, future research should focus on four aspects. First, researchers should quantitatively characterize the multi-factor coupling mechanism of flow pattern, water cut, CO_2_/H_2_S concentration, and sand content; clarify the critical threshold of phase inversion and corrosion acceleration; and establish targeted FAC prediction models. Second, researchers should optimize sensor layout, signal denoising, and feature extraction algorithms to improve the anti-interference ability and quantitative detection accuracy of mainstream NDT methods under high-temperature, high-pressure, and multiphase working conditions. Third, researchers should construct a multi-dimensional comparative evaluation system for NDT technologies based on the aspects of detection objects, applicable conditions, accuracy, and engineering cost to form a reasonable technical selection basis for actual pipeline engineering. Fourth, researchers should integrate NDT monitoring data with machine learning and digital twin technology to realize dynamic perception of FAC defect evolution and develop intelligent early-warning and full-life-cycle integrity management methods for multi-medium transmission pipelines.

## Figures and Tables

**Table 1:** Comparison of different corrosion forms of pipelines.

Corrosion Type	Core Definition	Electrochemical Mechanism	Key Influencing Factors
Flow-Accelerated Corrosion (FAC)	Corrosion intensified by the combined action of fluid flow and electrochemical reaction in multiphase flow pipeline systems	Fluid flow interacts with electrochemical processes, altering mass transfer and interface conditions to control corrosion rate	Flow velocity, flow pattern, wall shear stress, water cut, temperature and pressure
CO_2_ Corrosion	Internal corrosion of pipeline steel caused by dissolved carbon dioxide in aqueous medium	Carbonic acid formed by CO_2_ dissolution promotes hydrogen depolarization and accelerates anodic dissolution of steel	CO_2_ partial pressure, temperature, medium pH, pressure and water salinity
H_2_S Corrosion	Corrosion and hydrogen-induced embrittlement triggered by dissolved hydrogen sulfide in water phase	H_2_S ionization accelerates steel dissolution and induces hydrogen penetration into steel matrix	H_2_S partial pressure, temperature, pH, flow velocity and pipeline material hardness
Chloride-Induced Localized Corrosion	Local pitting and crevice corrosion induced by chloride ions penetrating surface protective films	Chloride ions break down passive films, forming occluded cells and triggering self-catalytic localized corrosion	Chloride concentration, dissolved oxygen, temperature, pH and surface film integrity
Microbiologically Influenced Corrosion (MIC)	Local corrosion accelerated by microbial metabolism and biofilm attachment on pipe inner surface	Microbial metabolites change local environmental conditions, synergistically promoting electrochemical corrosion	Microbial activity, biofilm structure, temperature, pH and dissolved oxygen level
Erosion-Corrosion	Combined material damage from particle mechanical erosion and electrochemical corrosion in multiphase flow	Solid particle impact damages surface films, exposing fresh metal and further aggravating electrochemical corrosion	Particle content, particle size, impact angle, flow velocity and medium corrosiveness

## Data Availability

No new data were created or analyzed in this study. Data sharing is not applicable to this article.
